# Identification of Insect-Deterrent Metabolites from *Acremonium masseei* strain CICY026, a Saprophytic Fungus from a Sinkhole in Yucatán

**DOI:** 10.3390/microorganisms7120712

**Published:** 2019-12-17

**Authors:** Ana L. Ruiz-Jiménez, Esaú Ruiz-Sánchez, Gabriela Heredia, Raúl Tapia-Tussell, Azucena González-Coloma, Karla Peraza-Jiménez, Felicia A. Moo-Koh, Irma L. Medina-Baizabal, Yanet Hernández-Romero, Gonzalo J. Mena-Rejón, Ramiro F. Quijano-Quiñones, Marcela Gamboa-Angulo

**Affiliations:** 1 Unidad de Biotecnología, Centro de Investigación Científica de Yucatán, A.C. Calle 43 x 32 y 34, No. 130, Col. Chuburná, Mérida, Yucatán 97205, Mexico; aruiz_ji@hotmail.com (A.L.R.-J.); kalicia11@hotmail.com (K.P.-J.); famk22@hotmail.com (F.A.M.-K.); baizabal@cicy.mx (I.L.M.-B.); 2 Tecnológico Nacional de México, Instituto Tecnológico de Conkal, Avenida Tecnológico s/n, C.P. Conkal, Yucatán 97345, Mexico; esau.ruiz@itconkal.edu.mx; 3 Red de Diversidad y Sistemática, Instituto de Ecología, A.C. Km 2.5 Carretera antigua a Xalapa-Coatepec No. 351, Xalapa 91070, Veracruz, Mexico; gabriela.heredia@inecol.mx; 4 Unidad de Energías Renovables, Centro de Investigación Científica de Yucatán, A.C. Calle 43 No. 130, Col. Chuburná, Mérida, Yucatán 97205, Mexico; rtapia@cicy.mx; 5 Departamento de Bioplaguicidas, Instituto de Ciencias Agrícolas, CSIC, Serrano 115-dpdo, 28006 Madrid, Spain; azu@ica.csic.es; 6 Investigación y Desarrollo, Bioactivos Agroquímicos de México, S. de R. L. de C. V. Ave. Río Pánuco 4345, Col. Campestre, Nuevo Laredo, Tamaulipas 88278, Mexico; yan1227@hotmail.com; 7 Facultad de Química, Universidad Autónoma de Yucatán, Calle 43 No. 163, Col. Inalámbrica, C.P. Mérida, Yucatán 97069, Mexico; mrejon@uady.mx (G.J.M.-R.); ramiro.quijano@correo.uady.mx (R.F.Q.-Q.)

**Keywords:** *Acremonium masseei*, acremonin A glucoside, antifeedant activity, *Bemisia tabaci*, hexahydroacremonintriol, *Myzus persicae*, *Rhopalosiphum padi*

## Abstract

Micromycetes from unexplored sources represent an opportunity to discover novel natural products to control insect pests. With this aim, a strain of *Acremonium masseei* CICY026 isolated from a tropical sinkhole was identified, cultured on fermented rice, and its ethyl acetate extract (EAE) was evaluated against three serious phytophagous insects (*Bemisia tabaci, Myzus persicae*, and *Rhopalosiphum padi*). DNA from *A. masseei* CICY026 was used to confirm its identity. EAE caused settling inhibition (SI) of *M. persicae* and *R. padi* (67.5% and 75.3%, respectively). Bioassay-guided fractionation of the active EAE led to the isolation of a novel metabolite, named hexahydroacremonintriol (**1**), and of acremonin A glucoside (**2**). The structures of **1** and **2** were determined using IR, one- and two-dimensional NMR, HRMS, and confirmed by theoretical data. The aphid *M. persicae* was noticeably sensitive to **1** and **2** (SI: 55.6% and 67.2%, respectively), whereas *R. padi* was only slightly affected by **1** (SI: 59%). This new knowledge about mycobiota from these special sinkhole ecosystems will inform the development of new biorational pesticides.

## 1. Introduction

Among the insect pests that limit horticultural crop production, the sap-feeding whitefly *Bemisia tabaci* Genn. (Hemiptera: Aleyrodidae), green peach aphid *Myzus persicae* Sulzer (Hemiptera: Aphididae), and bird cherry-oat aphid *Rhopalosiphum padi* Linn. (Hemiptera: Aphididae) are three of the most damaging to vegetable crops. Their direct feeding and transmission of viruses into Cucurbitaceae, Fabaceae, and Solanaceae crops can cause severe damage [1,2,3,4].

**Figure 1:**
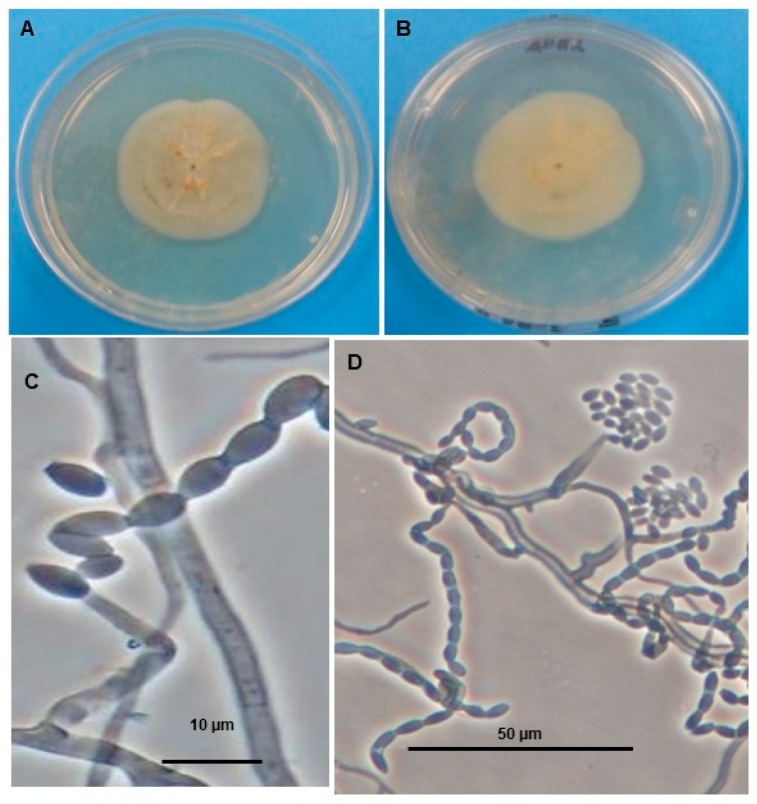
*Acremonium masseei* on potato dextrose agar (PDA) plate. (**A**) Top side and (**B**) reverse side of colony, (**C**) doliform conidia, (**D**) conidia forming chains, spirally arranged, and grouped in mucilaginous heads.

As part of our institutional program to search for new natural alternatives to manage agricultural pests, we previously isolated fungal strains from the Yucatán Peninsula and reported a high antifeedant effect of a mixture of fatty acids from *Gliomastix masseei* strain CICY029 on *Myzus persicae* and *Rhopalosiphum padi* [5]. During our screening of fungi for possible novel metabolites with biological activity, we have also studied *Acremonium masseei* strain CICY026 (Figure 1), a micromycete isolated from plant debris submerged in a sinkhole, a typical freshwater ecosystem in the peninsular region of Yucatán [6].

*G. masseei* is recognized as belonging to the genus *Acremonium*, which comprises about 212 species [7,8]. Tian [9] reported 356 metabolites that had diverse biological activities from *Acremonium* species, but little is known about the insecticidal activity of *Acremonium* metabolites. Peramine from *Acremonium loliae* is active against adults and larvae of *Listronotus bonariensis* [10], cephaibol A from *Acremonium tubakii* is active against the ectoparasite *Cimex lectularius* [11], and chloramphenicol derivatives from *Acremonium vitellinum* are active against *Helicoverpa armigera* [12]. 

Therefore, here we molecularly characterized CICY026 strain of *A. masseei* in a detailed analysis of its DNA fingerprint sequence. We also isolated insect-deterrent compounds from an ethyl acetate extract (EAE) of the fungus and identified active compounds using one- and two-dimensional NMR.

## 2. Materials and Methods

### 2.1. General

Fungal morphological characters were measured using a light microscope at 1000× and photomicrographs were captured with a Nikon DS-L3 camera adapted to a Nikon 80i microscope (Nikon Corp. Mitsubishi, JP). PCR products were purified and sequenced by Macrogen (Seoul, Korea). DNA was amplified using a GeneAmp 9700 DNA Thermal Cycler (PerkinElmer, Life Technology, Carlsbad, CA, USA).

The IR spectrum was recorded with a Nicolet 8700 FTIR spectrometer (Thermo Electron, Madison, WI, USA). Gas chromatography–mass spectrometry (GC-MS) analyses were done with an Agilent Technologies (Santa Clara, CA, USA) 6890N Chromatograph coupled to an Agilent 5975B mass-selective detector as previously described [5]. Low- and high-resolution MS spectra were recorded on a GC Mate II mass spectrometer in FAB [+] mode using NBA matrix and JEOL Calibration Ultramark and Resolution 3000 (JEOL, Peabody, MA, USA). ^1^H and ^13^C NMR spectra were recorded in an AMX-400 spectrometer (Bruker Corp., Billerica, MS, USA) at 400 and 100 MHz, respectively, using TMS as an internal standard, and in a Varian, Agilent AR Premium Compact (Varian, Palo Alto, CA, USA) at 599.774 and 150.826 MHz, respectively. Chemical shifts were reported in ppm and coupling constants (*J*) were given in Hz. Two-dimensional experiments (COSY, DEPT, HSQC, and HMBC) were done using the Varian equipment.

### 2.2. Fungal Material 

The fungus *A. masseei* CICY026 (Figure 1) was obtained from the culture collection of the Unidad de Biotecnología, Centro de Investigación Científica de Yucatán (CICY). This fungus was isolated from plant litter submerged in a sinkhole in Mérida, Yucatán [6].

The fungus was reactivated on commercial potato dextrose agar (PDA, Dibico, Edo. Mex., MX) at 25 ± 2°C with a 12 h light–12 h dark period for 7 d in an incubator (Precision Scientific, Buffalo NY, USA). The mycelial mat and conidia were either frozen and lyophilized (Labconco FreeZone 2.5, model 7670520, Houston, TX, USA) for molecular studies or kept as agar slant cultures at 5 °C (8–10 months) or in 10% glycerol at −80 °C, or prepared as a conidial–hyphal fragments suspension.

*Gliomastix masseei* CICY029 strain was isolated from plant debris in Dzibilchaltun archeological zone and cultured as described previously by Reyes-Estebanez et al. [13].

### 2.3. Molecular Identification of Fungal Strains

#### 2.3.1. Isolation of DNA

Total genomic DNA was extracted according to the method of Moo-Koh et al. [14], spectrophotometrically quantified, and quality determined as previously described [15]. Sequences were edited to eliminate noise at ends, minus strands were converted into reverse complement, and both strands were aligned. The 500 bp sequence was deposited in GenBank as accession KY171948.

#### 2.3.2. PCR Amplification and Sequencing of 5.8S-ITS of rDNA

The identity of the isolated *G. masseei* CICY026 and CICY029 strains was corroborated through sequence analysis of the 5.8S-ITS regions with universal primers ITS1 and ITS4 [15,16]. DNA was sequenced in both directions to ensure that there was no misreading. PCR products were purified and sequenced by Macrogen. Sequences were aligned and edited using the BioEdit program v 7.0.5 (Ibis therapeutics, CA, USA) [17] and visually corrected. Sequences were then compared against those available in the GenBank database. The sequences were aligned, and a tree was constructed with the Mega 7 program (Tamura, PA, USA) [18].

### 2.4. PCR Fingerprint Analyses 

#### 2.4.1. Directed Amplification of Minisatellite DNA (DAMD) Reactions

PCR-based fingerprinting was performed using four minisatellite core sequence primers for directed amplification of minisatellite DNA (DAMD) as done previously [19]. PCR reactions were performed in 25 µL reaction mixture containing 1× PCR buffer (10× 200 mM Tris-HCl, 500 mM KCl, pH 8.4) (Invitrogen by Thermo Fisher Scientific, Carlsbad, CA, USA), 0.2 mM of each dNTP (Invitrogen), 2 mM MgCl_2_, 0.2 µM primers, 60 ng of template DNA, and 1 U of Taq polymerase (Invitrogen by Thermo Fisher Scientific, Carlsbad, CA, USA). DNA was amplified using a GeneAmp 9700 DNA Thermal Cycler (PerkinElmer, Life Technology, Carlsbad, CA, USA) with an initial denaturation at 95 °C for 5 min; 40 cycles of 1 min at 95 °C, 2 min at 55 °C, and 2 min at 72 °C; and a final extension of 5 min at 72 °C. Amplified DNA fragments were separated by electrophoresis in a 1.5% (*w/v*) agarose gel and visualized after ethidium bromide staining under a UV transilluminator. The images were taken with a UVP BioImaging System (UVP, Inc., Upland, CA, USA).

#### 2.4.2. Arbitrarily Primed PCR (AP-PCR) 

For PCR-based fingerprinting, five 15 bp minisatellite primers (MSP) were used according to previous reports [19,20]: for (TCC)_5_ and (GACAC)_3_, the annealing temperature was 42 °C [20]; for (GAC)_5_, (CAG)_5_, and (GTG)_5_ the annealing temperature was 60 °C [21]. PCR amplification was performed in a 25 µL reaction mixture containing 1× PCR buffer (10× 200 mM Tris-HCl, 500 mM KCl, pH 8.4; Invitrogen), 0.25 mM each dNTP (Invitrogen, by Thermo Fisher Scientific, Carlsbad, CA, USA), 2 mM MgCl_2_, 0.8 µM primers, 10 ng of template DNA, and 1 U of Taq polymerase (Invitrogen, by Thermo Fisher Scientific, Carlsbad, CA, USA). DNA amplification was performed using a GeneAmp 9700 DNA Thermal Cycler (PerkinElmer, Life Technology, CA, USA), with an initial denaturation at 95 °C for 5 min; 40 cycles of 40 s at 95 °C, 60 sec at the appropriate annealing temperature, and 60 sec at 72 °C; and final extension of 5 min at 72 °C. The amplified DNA fragments were separated by electrophoresis in 1.5% (*w/v*) agarose gels and visualized after ethidium bromide staining. A molecular size marker (1 kb Plus DNA Ladder, Invitrogen) was added to each gel as a reference.

### 2.5. Fungal Extraction and Purification of Compounds

For the production of the mycelia and its metabolites, fermented rice was used. Rice (Valle Verde, MX) was fermented in distilled water (20 g/30 mL, each bottle, total 30 bottles) at room temperature overnight, then sterilized at 121 °C for 30 min [13]. After 24 h, each bottle of fermented rice was inoculated with 1 mL of a conidial–hyphal fragments suspension (vide supra) from a PDA seed culture of *A. masseei* CICY026 as previously described [6]. After 40 d, the mycelium was lyophilized and stored at 4 °C in the dark until used.

The lyophilized mycelium was manually fragmented and successively extracted with ethyl acetate (three times, 50 mL, 24 h each extraction) at room temperature. The solvent was vacuum-evaporated in a rotary evaporator (RV 10 Control, IKA, Staufen, Germany), yielding 3.7 g of crude extract (6.1% yield). This crude ethyl acetate extract (EAE) was partitioned using acetonitrile and hexane (three times, 2:1, 1:1, 1:1, v/v) into fractions 1A and 1B, respectively. The 1A fraction with medium polarity compounds was separated using silica gel (Sigma Aldrich, St. Louis, MO, USA) packed in a glass column chromatography, eluted with mixtures of hexane, acetone, and methanol, and monitored using TLC (Sigma Aldrich, St. Louis, MO, USA) to produce 16 subfractions. Subfractions 11 and 12 were combined and subjected to silica gel (Sigma Aldrich, St. Louis, MO, USA) packed in a glass column chromatography and eluted with a mixture of dichloromethane, acetone, and methanol (75:20:5) to yield compounds **1** (5 mg) and **2** (10 mg). All samples were stored at 4 °C in the dark until used.

### 2.6. Computational Details

Full geometry optimizations of the structure of compound 1 were carried out using DFT at the M06-2X/6-311++G(2d,2p) level. The nature of all the stationary points was determined by computing and diagonalizing the Hessian matrix at the same level; no imaginary frequencies were presented in the final geometry. All the structural optimizations were performed with Gaussian 09 [22]. The isotropic shielding constant (σ) in the corresponding M06-2X/6-311++G(2d,2p) geometries was calculated at the ωB97X-D/6-31G* level and corrected using a scheme involving topology and bond orders with neighboring atoms and Spartan16 [23] software.

### 2.7. Insecticidal Activity

#### 2.7.1. Bioassay on Oviposition Inhibition on *Bemisia tabaci*

Adult whiteflies were obtained from a stock colony of *B. tabaci* maintained on eggplant plants and kept in a greenhouse at 25–35 °C, 55–75% relative humidity, and natural light [24].

For the choice bioassay, discs of fresh habanero pepper leaves (2 cm^2^) were cut in half, set separately on water agar (2% *w/v*), and placed in the lid of a 10 mL plastic jar. Previously, samples (10 mg/mL) were dissolved with dimethylsulfoxide:water (1:1, v/v). Each half leaf was treated (*T*) with 10 µL of a fungal extract dissolved (100 µg/cm^2^), and the other half was treated with dimethylsulfoxide:water (1:1, 10 µL) as a control (*C*). Once the leaves were treated, 30 adult whiteflies were placed in a plastic jar that was set upside down, so that the treated and control leaf sections were on the bottom. Jars were incubated upside down at 26 ± 2 °C, 75% ± 8% relative humidity, and natural light. An ethyl acetate extract of fermented rice was used as the blank. Five replicates for each extract were tested. The repellent activity of the extracts was assessed as oviposition inhibition based on the number of eggs laid on the leaf discs 48 h after *B. tabaci* exposure. Oviposition inhibition (% OI = [1− (*T*/*C*) × 100]) was calculated for the EAE and fractions at an initial dose of 100 µg/cm^2^, and for the pure compounds at 50 µg/cm^2^, where *T* is the number of eggs on the treated surface and *C* is the number of eggs on the control surface [25].

#### 2.7.2. Bioassay of Settling Inhibition of *Myzus persicae* and *Rhopalosiphum padi*

Adults of *M*. *persicae* and *R*. *padi* were grown on pepper (*Capsicum annuum* L.) and barley (*Hordeum vulgare* L.) plants, respectively. Colonies were maintained at 21 ± 2 °C, 60–70% relative humidity, and 16 h light:8 h dark in a growth chamber [26].

For the bioassay, two fragments each of pepper and barley (1 cm^2^) leaves were treated with the organic extract and isolated compounds (100 µg/cm^2^) or only the solvent (acetone). Treated leaf sections were set on water agar (2%) coating the bottom of plastic boxes (3×3×1.5 cm). In this experiment, 20 replicates per extract were performed. After solvent evaporation, 10 apterous aphids were taken from the colony and placed in each plastic box. The percentage of aphids that settled on each leaf section was recorded after 24 h as described by González-Coloma [26]. A settling inhibition index (% SI) was calculated for the organic extract and isolated compounds at an initial concentration of 100 and 50 µg/cm^2^, respectively [% SI = 1 − (% *T*/% *C*) × 100, where % *T* is the percentage of aphids on a treated surface and % *C* is the percentage of aphids on a control surface].

## 3. Results and Discussion

### 3.1. Molecular Identification of *Acremonium masseei* Strain CICY026

**Figure 2:**
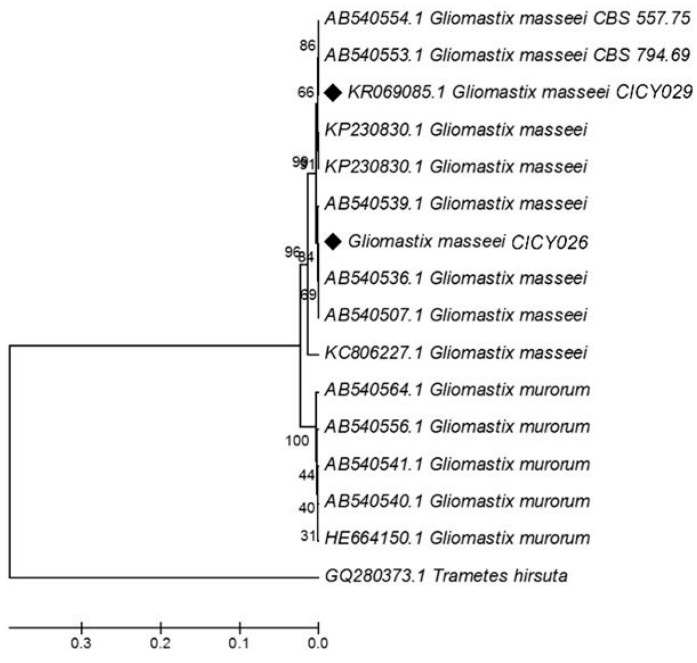
UPGMA phylogram showing the grouping of various polymorphic sequences of the 5.8S-ITS regions of rDNA from *Gliomastix masseei* CICY026 and CICY029. Bootstrap percentages are shown at the branches and were determined from 1000 iterations.

**Figure 3:**
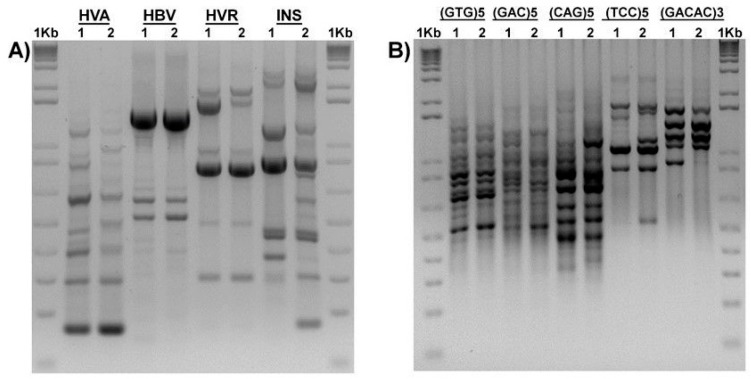
Fingerprints obtained with different mini-microsatellite primers. (**A**) Primers DAMD (directed amplification of minisatellite DNA); (**B**) microsatellite primers (MSP). 1: *Acremonium masseei* CICY026, 2: *Gliomastix masseei* CICY029.

*A. masseei* strain CICY026 was molecularly identified, and its sequence deposited in GenBank (accession KY171948). DNA from fungal isolates CICY026 and CICY029 was used in the PCR with the universal primers ITS1 and ITS4 for amplification of the ITS region, including the 5.8S rRNA gene. A PCR product of approximately 600 bp was amplified from the two strains. Sequence analysis of the ITS region from the two strains revealed 99% homology with DNA sequence from *G. masseei* strain CICY029. In the UPGMA tree constructed in the phylogenetic analysis to confirm the identity of these strains (Figure 2), both strains were grouped in the same clade with the *G. masseei* accessions from the GenBank database, but in a different subclade, indicating that they are different isolates. Therefore, the fingerprint of both strains using 15 bp minisatellite primers (MSP) was confirmed (Figure 3).

### 3.2. Isolation and Structural Identification of Compounds 1 and 2 

**Figure 4:**
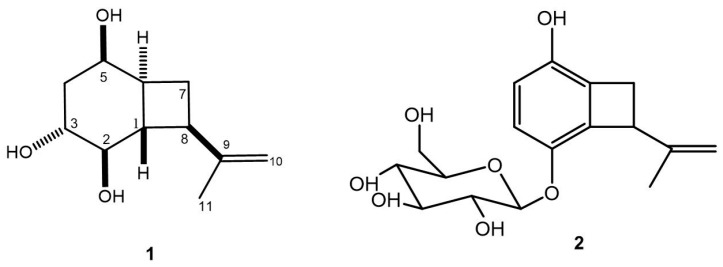
Compounds produced by *Acremonium masseei* CICY026. **1**) Hexahydroacremonintriol, **2**) acremonin A glucoside.

The EAE from *A. masseei* was partitioned, and the defatted fraction was eluted through the chromatography columns of silica gel, to yield a new compound named hexahydroacremonintriol (**1**) and the known acremonin A glucoside (**2**). This last was identified by comparison of its ^1^H and ^13^C NMR data with the literature [27] (Figure 4, Figure S1).

Compound **1** was obtained as a colorless oil; the molecular formula was determined by HRFABMS as C_11_H_18_O_3_, with an *m/z* value of the molecular ion of 199.1330 (calc. for *m/z* 199.1334 [M + H]^+^) with three degrees of unsaturation. The structure was deduced from analysis of ^1^H, ^13^C NMR, and 2D-NMR spectral data (Table 1, Figure S1). 

**Figure 5:**
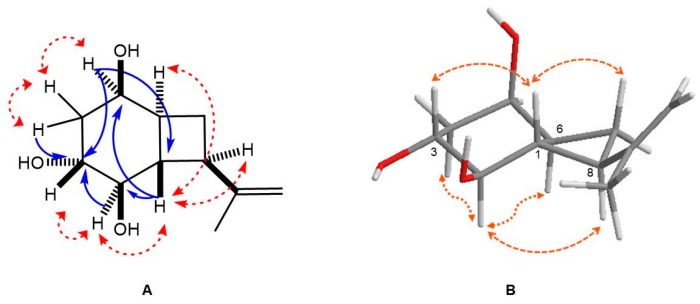
(**A**) Selected HMBC (blue) and COSY (red) correlations for compound **1**, (**B**) M06-2X/6-311++G(2d,dp) optimized structure for compound **1**, and NOESY correlations.

The expected 11 carbons were observed in the ^13^C NMR and DEPT spectra for compound **1** as only one quaternary sp^2^ carbon (147.3 ppm), six methane groups (three oxygenated at 78.9, 72.7, 66.6 ppm), three methylene (one sp^2^ at 109.3 ppm) and only one methyl group (21.0 ppm) were found. The only double bond in the structure of **1** was assigned to an isopropenyl chain, also observed in compound **2,** with olefinic methylene protons (4.80 and 4.74 ppm) and a vinylic methyl group (1.72 ppm) in its ^1^H NMR spectrum. Subsequently, a saturated bicyclic molecule explained the other two degrees of unsaturation. These data with the rest of the carbons (Table 1) suggested a fused cyclobutane and cyclohexane rings, very similar to the aglucone part of metabolite **2** [27]. On the other hand, the alcohol groups in the structure of **1** detected in its IR spectrum as a strong band at 3382 cm^−1^ (IR ν_max_ [KBr]: 3382, 2925, 2859, 1483, and 1247 cm^−1^) was confirmed by the presence at low field of three methine protons shifted at 4.04, 3.73, and 3.49 ppm in its ^1^H NMR spectrum. These alcohol groups were placed in the cyclohexane ring, two of them were located in position C-2, and C-5 based on the COSY and HMBC data and comparison with **2** (Figure 5A). Vicinal couplings between methylene protons H-4a, H-4b with methine protons H-3 and H-5, and H-1/H-3 protons were easily discerned in the COSY analysis. Long-range correlations (*^2^J* and *^3^J*) of the HMBC experiment of **1** showed correlations for proton H-5 with C-1, C-3, and C-4, and for proton H-1 with oxygenated C-2 and C-5. The additional hydroxyl group relative to **1** was placed at C-3 according to the long-range (*^2^J*) correlation between H-2, H-4a, and H-4b. The saturated bicyclic molecule is slightly similar to *bis*-homoinositol, a synthetic derivative of inositol [28]. 

In addition, the double of doublet of doublets at 2.04 ppm, with coupling constants of 10.2, 10.2, and 11.8 Hz, assigned to proton H-1 revealed the presence of three vicinal protons, each one in *trans* orientation (H-2, H-6, and H-8). Therefore, the structure of compound **1**, showing the *trans* arrangement between H1 and H-2, H-6, and H-8, was optimized (Figure 5B), and the corresponding dihedral angles were calculated. The values of the dihedral angles were 178.5°, 171.7°, and 169.4° for H-5/H-4, H-5/H-6, and H-5/H-8, respectively. These values match the *trans* arrangement revealed for coupling constants, suggesting a *trans*-fused bicyclic molecule as found in the structure of punctaporonin and analog metabolites [29,30,31]. 

The relative configuration of **1** was deduced using a NOESY analysis, which displayed key spatial couplings between H-2 with H-4b, H-6, H-7b, and H-8, which led us to locate these protons on the same face of the structure. By contrast, the correlations of protons H-1 with H-3 and H-7a were located on the opposite face (Figure 5B). The structure of **1** was then identified as (1S*,2R*,3R*,5R*,6S*,8R*)-8-(prop-1-en-2-yl) bicyclo[4.2.0]octane-2,3,5-triol with the trivial name of hexahydroacremonintriol (Figure 4). 

To confirm the proposed structure of compound **1**, we calculated the ^13^C chemical shifts at the wB97X-D/6-31G* level on the M06-2X/6-311++G(2d,dp) optimized geometry (Figure 5B), using the corrected model as implemented in the Spartan16 software. The results were compared with the measured experimental ones. The root mean squares (rms) of the differences between experimental and chemical shifts were calculated. The results are in Table 2.

The small value of the rms clearly indicated excellent agreement between the experimental values of the chemical shifts and the calculated, confirming the spatial arrangement proposed for the hexahydroacremonintriol (Figure 5B).

Compound **2** had a glucoside ring linked to an alcohol in position C-5 of the phenolic ring, and together compounds **1** and **2** might be biosynthesized by a common precursor, or by microbial oxidation of the aromatic precursor acremonin A [27,28,32]. This is the second report of compound **2**, also isolated from a fungus of *Acremonium* genus.

### 3.3. Insecticidal Activity of *Acremonium masseei* CICY026

**Figure 6:**
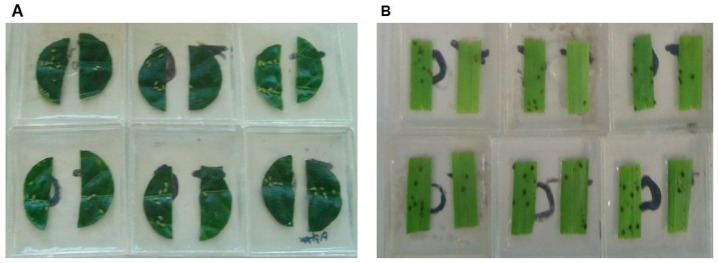
Antifeedant effect of ethyl acetate extract from *Acremonium masseei* on adults of (**A**) *Myzus persicae* and (**B**) *Rhopalosiphum padi*.

The results of the evaluations of EAE, fractions and compounds obtained from *A. masseei* on the phytophagous *B. tabaci*, *M. persicae*, and *R. padi* are shown in Table 3. The greatest inhibitory effect was found with 100 µg/cm^2^ EAE on the settling of *M. persicae* and *R. padi*. When the extract was separated into acetonitrile and hexane fraction, both fractions had a similar effect only on *M. persicae*. Purified compound **1** (50 µg/cm^2^) inhibited settling of both aphid species (SI = 55.6% and 67.2%, respectively), whereas compound **2** only inhibited settling of *M. persicae* (SI = 59%). Neither compound had any activity against *B. tabaci* (Table 3, Figure 6). 

The *A. masseei* and *G. masseei* strains were both collected in the state of Yucatán (México). However, *A. masseei* CICY026 was isolated from a submerged leaf in freshwater ecosystem (cenote X´kan ho ho che) [6], while *G. masseei* CICY029 was isolated from plant debris on soil [5]. This is the second report of this species in Yucatán state. Fungi from the genus *Acremonium* have been isolated from both terrestrial and marine sources [8]. Diverse species belonging to the *Acremonium* genus have been recognized as rich sources of biologically active metabolites against human and agricultural pathogens. The 356 metabolites from *Acremonium* genus reported by Tian [10] have effects such as anti-inflammatory, antimalarial, antimicrobial, antioxidant, antitumor, antiviral, cytotoxic, immunosuppressive, neuritogenic, phytotoxic, tremorgenic, inhibitory to enzymes, but only one compound was active against insects.

The hexane fraction had activity only against *M. persicae*, which we attribute to the presence of unsaturated fatty acids detected in the GC-MS chromatogram of the hexane fraction. Fatty acids such as linoleic and linolenic acids have previously been reported to have an anti-settling activity against *M. persicae* and *R. padi* [5,33,34,35].

Acremonin A glucoside from *Acremonium* sp. (related *Acremonium roseogriseum*), a fungus isolated from a marine alga [27], has been well characterized as an antioxidant, but has not been reported as an insect deterrent. Its activity may depend on the number and location of the hydroxyl groups, suggesting that hydroxyl groups contribute to the deterrent activity against different pests [36]. The deterrent activity also depends on the species of insects. Strasser [37] reported that *M. persicae* is susceptible to destruxin E (LD_50_: 0.4 µg/cm^2^), but *R. padi* feeds on leaves treated with destruxin E, even at relatively high doses (e.g., 6.6 mg/cm^2^).

Even though *A. masseei* CICY026 and CICY029 strains were isolated from relatively close sites, the environmental conditions differed at the sites; hence, their chemical and biological profiles differed. 

## 4. Conclusions

Scarcely explored environments such sinkholes are a rich source of microorganisms with biotechnological potential. The EAE and fractions of the fungus *A. masseei* CICY026 cultured on fermented rice had deterrent activity against three phloem-feeding insect pests, *Bemisia tabaci, Myzus persicae*, and *Rhopalosiphum padi*. The active fraction contained the novel compound hexahydroacremonintriol and second report of the known metabolite acremonin A glucoside. Novel fungal metabolites with biological activity against pest insects are valuable agents for developing new biorational pesticides. These might be used directly as active ingredients or by using them as lead compounds. Further greenhouse and field evaluations of these compounds are required to determine the potential of these metabolites for pest management in realistic scenarios.

## Figures and Tables

**Table 1:** NMR data for hexahydroacremonintriol (**1**) recorded in CDCl_3_ (^1^H and ^13^C NMR at 600 and 150 MHz, respectively).

Position	^13^C ppm	δ ^1^H, m, *J* = Hz	COSY	HMBC
1	43.5 (d)	2.04, ddd, 10.2, 10.2, 11.8	2, 6, 8	C-2, C-5
2	78.9 (d)	3.49, dd, 8.2, 10.5	1, 3	C-3
3	72.7 (d)	3.73, ddd, 5.1, 8.2, 11.4	2, 4a, 4b	
4	41.7 (t)	a. 2.14, ddd, 2.6, 5.0, 13.9	3, 4b, 5	C-3
		b. 1.59, ddd, 2.8, 11.4, 14.0	3, 4a, 5	C-3
5	66.6 (d)	4.04, dd, 2.5, 5.0	4a, 4b	C-1, C-3, C-4
6	41.3 (d)	1.75, m	1, 5, 7a, 7b	C-8, C-5
7	28.6 (t)	a. 1.62, ddd, 9.2, 9.2, 10.9	6, 7b, 8	
		b. 1.99, ddd, 6.4, 9.2, 12.9	6, 7a, 8	
8	46.4 (d)	2.54, bdd, 9.2, 15.8	1, 7a	
9	147.3 (s)			
10	109.3 (t)	a. 4.80, bs	8, 10b, 11	C-8, C-9, C-11
		b. 4.74, dd, 1.4, 2.9	10a, 11	C-8, C-9, C-11
11	21.0 (q)	1.72, s	10a, 10b	C-8, C-9, C-10

**Table 2:** Differences between ^13^C NMR experimental chemical shifts and the calculated for the ωB97X-D/6-31G*-corrected model for compound **1.**

Carbon No.	δ ^13^C	
	Experimental	Theoretical
C1	66.6	65.7
C2	41.7	41.0
C3	72.7	72.8
C4	78.9	78.9
C5	43.5	44.9
C6	41.3	40.5
C7	28.6	24.9
C8	46.4	44.9
C9	147.3	144.6
C10	109.3	110.3
C11	21.0	21.1
**rms**		**1.60**

**Table 3:** Repellent activity of the crude extract, fractions (100 µg/cm^2^) and pure compounds (50 µg/cm^2^) of *Acremonium masseei* production on *Bemisia tabaci*, *Myzus persicae,* and *Rhopalosiphum padi* for 24 h exposure.

Sample	% OI *B. tabaci*	% SI *M. persicae*	% SI *R. padi*
EtOAc extract	35.7 ± 11.4	67.5 ± 7.4	75.3 ± 5.0
Fraction 1A (AcN fraction)	31.6 ± 10.7	76.1 ± 5.8	48.6 ± 6.9
Fraction 1B (hexane fraction)	43.8 ± 14.8	67.8 ± 7.0	41.5 ± 7.5
**1**	24.4 ± 8.6	55.6 ± 5.4	59.0 ± 4.5
**2**	19.6 ± 6.2	67.2 ± 4.8	48.1 ± 4.9
Blank (fermented rice)	34.1 ± 12.8	34 ± 8.2	48.4 ± 7.3

**%** OI: oviposition inhibition; **%** SI: settling inhibition.

## Supplementary Materials

The following are available online at https://www.mdpi.com/2076-2607/7/12/712/s1, Figure S1: (**A**) NMR spectra of compound **1**, (**B**) ^1^H and ^13^C spectra of compound **2**.
