# Supplementary material for: Identification of Insect-Deterrent Metabolites from Acremonium masseei strain CICY026, a Saprophytic Fungus from a Sinkhole in Yucatán
Source: Microorganisms. 2019 Dec 17;7(12):712. doi: 10.3390/microorganisms7120712 (PMC6955848; doi:10.3390/microorganisms7120712)
Supplement: Supplementary file 1 [file microorganisms-07-00712-s001.pdf]

Identification of Insect-Deterrent Metabolites from *Acremonium masseei* strain CICY026, a Saprophytic Fungus from a Sinkhole in Yucatán

Ana L. Ruiz-Jiménez[1], Esaú Ruiz-Sánchez[2], Gabriela Heredia[3], Raúl Tapia-Tussell[4], Azucena González-Coloma[5], Karla Peraza-Jiménez[1], Felicia A. Moo-Koh[1], Irma L. Medina-Baizabal[1], Yanet Hernández-Romero[6], Gonzalo J. Mena-Rejón[7], Ramiro F. Quijano-Quiñones[7] and Marcela Gamboa-Angulo[1,*]

[1] Unidad de Biotecnología, Centro de Investigación Científica de Yucatán, A.C. Calle 43 x 32 y 34, No. 130, Col. Chuburná, Mérida, Yucatán, 97205, México. E-Mail: aruiz_ji@hotmail.com (A.L.R.-J.); kalicia11@hotmail.com (K.P.-J.); famk22@hotmail.com (F.M.-K.); baizabal@cicy.mx (I.L.M.-B.); mmarcela@cicy.mx (M.G.-A.)

[2] Tecnológico Nacional de México. Instituto Tecnológico de Conkal. Avenida Tecnológico s/n, C.P. 97345. Conkal, Yucatán, México; E-mail: esau.ruiz@itconkal.edu.mx (E.R.-S.)

[3] Red de Diversidad y Sistemática, Instituto de Ecología, A.C. Km 2.5 Carretera antigua a Xalapa-Coatepec No. 351, Xalapa 91070, Veracruz, México. E-Mail: gabriela.heredia@inecol.mx (G.A.)

[4] Unidad de Energías Renovables, Centro de Investigación Científica de Yucatán, A.C. Calle 43 No. 130, Col. Chuburná, Mérida, Yucatán, 97205, México. E-Mail: rtapia@cicy.mx (R.T.-T.)

[5] Departamento de Bioplaguicidas, Instituto de Ciencias Agrícolas, CSIC. Serrano 115-dpdo. Madrid, 28006, España. E-Mail: azu@ica.csic.es (A.G.-C.)

[6] Investigación y Desarrollo, Bioactivos Agroquímicos de México, S. de R. L. de C. V. Ave. Río Pánuco 4345, Col. Campestre, Nuevo Laredo, Tamaulipas, 88278, México. E-Mail: yan1227@hotmail.com (Y.H.-R.)

[7] Facultad de Química, Universidad Autónoma de Yucatán. Calle 43 No. 163, Col. Inalámbrica, C.P. 97069 Mérida, Yucatán, México. E-Mail: ramiro.quijano@uady.mx (R.F.Q.-Q.); mrejon@correo.uady.mx (G.J.M.-R.)

*Correspondence: E-mail: mmarcela@cicy.mx (M.G.-A.). Tel. +52-999-981-3923; Fax +52-999-981-3900

Contents:

A. Compound **1** NMR spectra: 1. Proton, 2. Carbon, 3. COSY, 4. HSQC, 5. HMBC and 6. NOESY

B. Compound **2** NMR spectra: 1. Proton and 2. Carbon

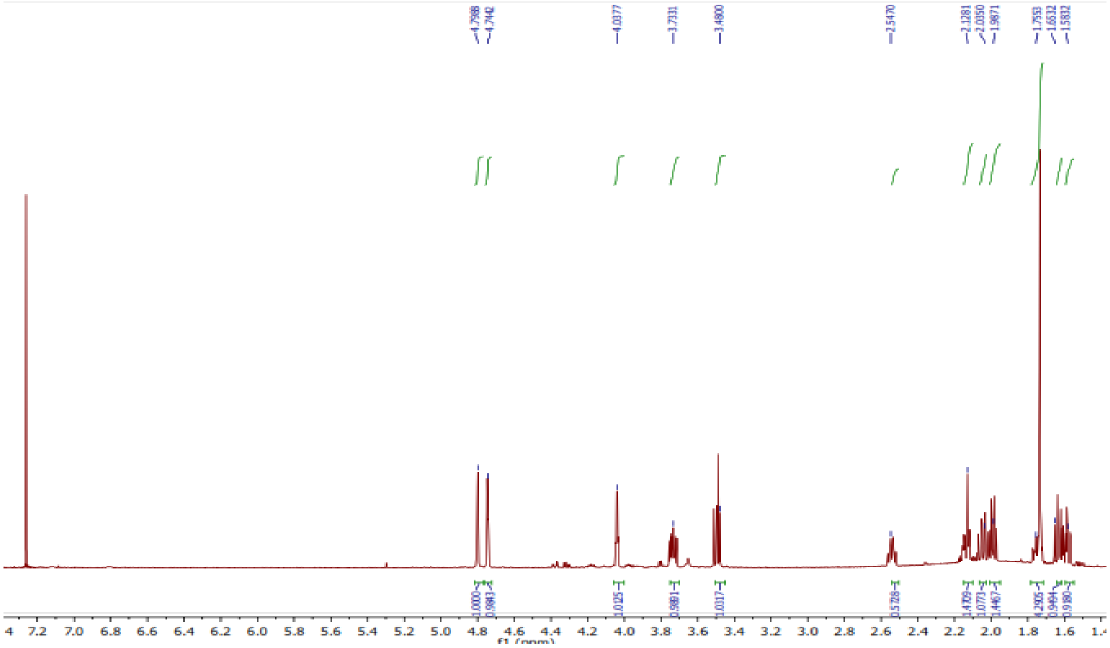

A1. ^{1}H spectrum of compound **1** ($CDCl_3$, 600 MHz)

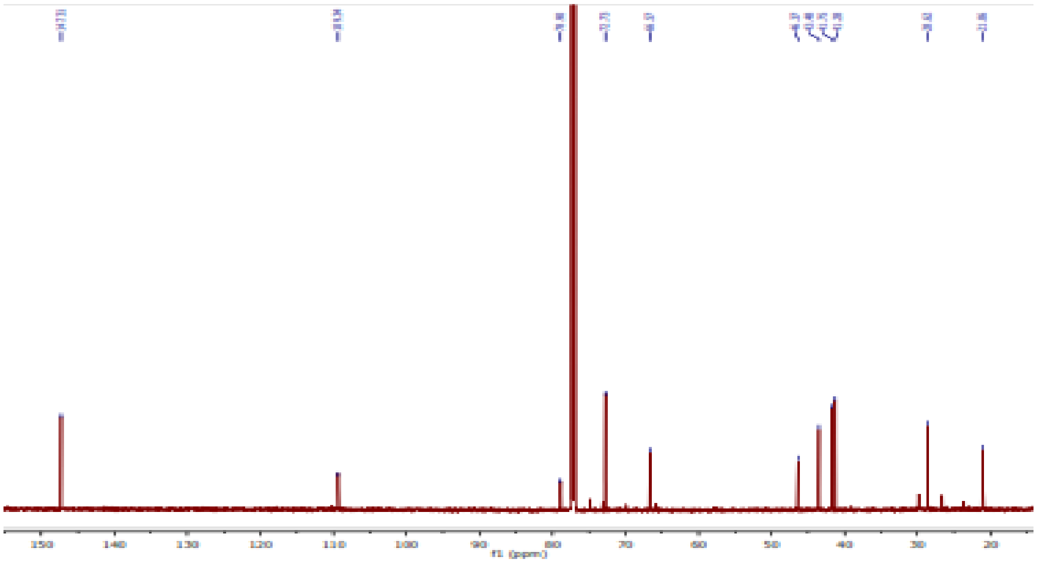

A2. ^{13}C spectrum of compound **1** ($CDCl_3$, 150 MHz)

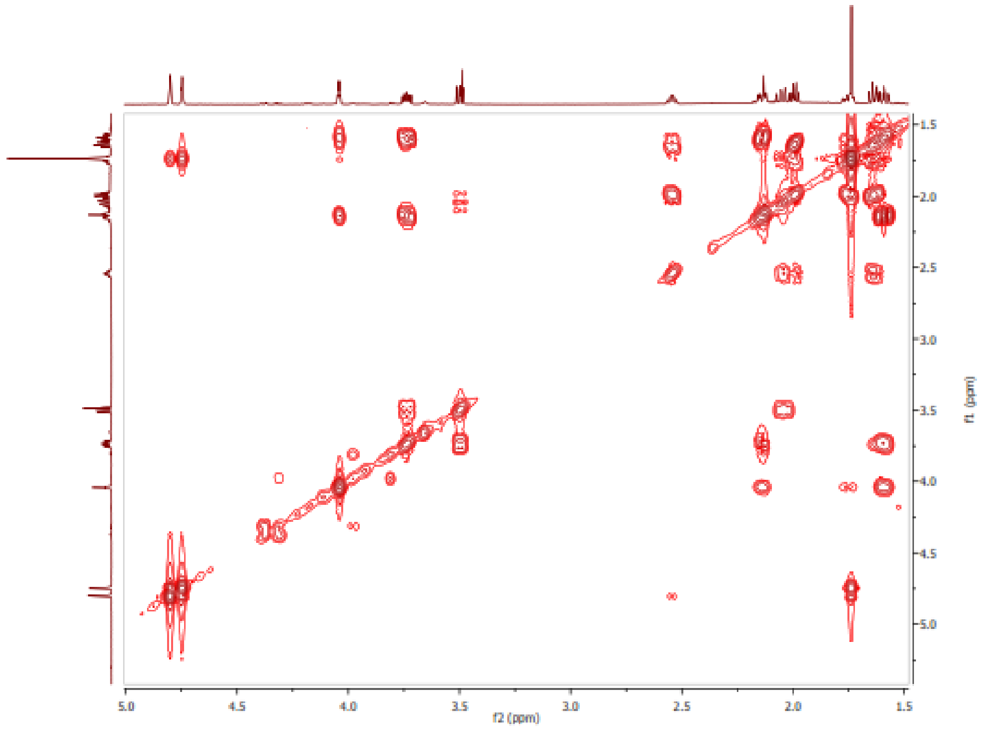

A3. ^{1}H ^{1}H-COSY spectrum of compound **1** ($CDCl_3$, 600 MHz)

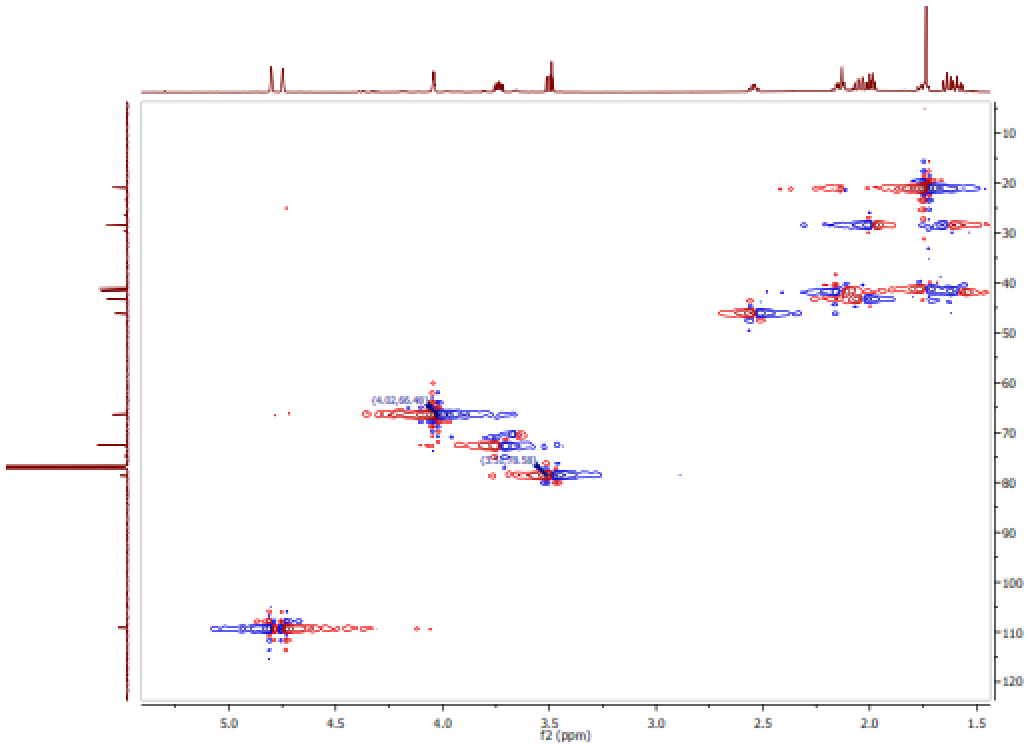

A4. HSQC spectrum of compound **1** ($CDCl_3$)

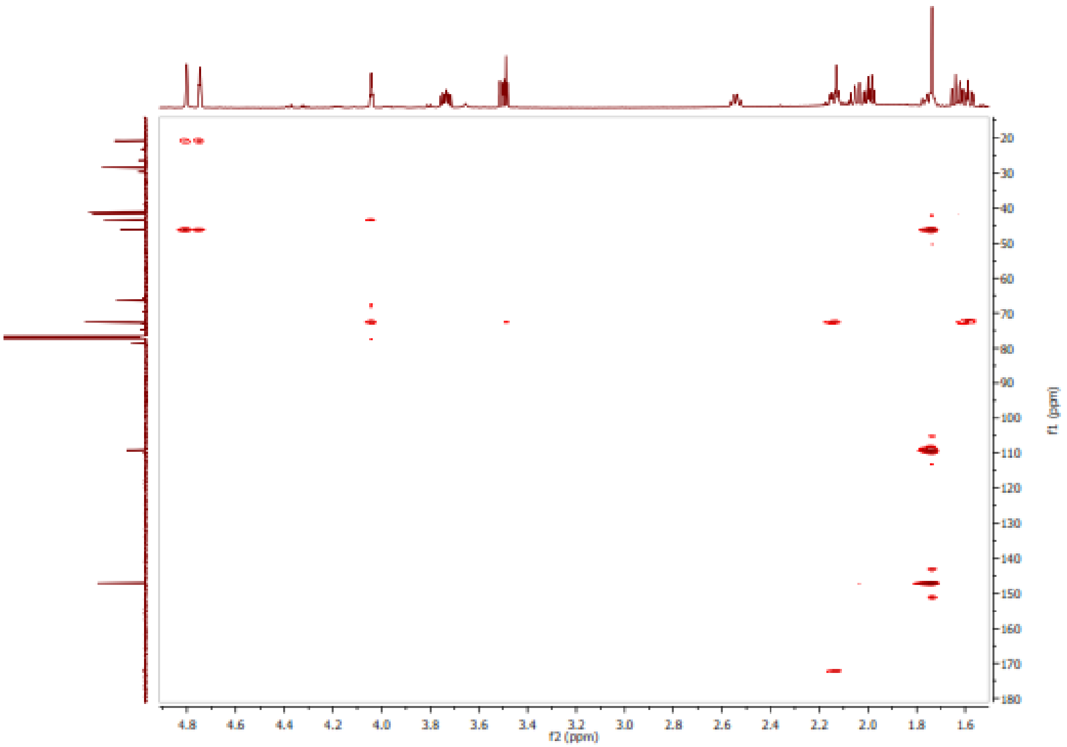

A5. HMBC spectrum of compound **1** ($CDCl_3$)

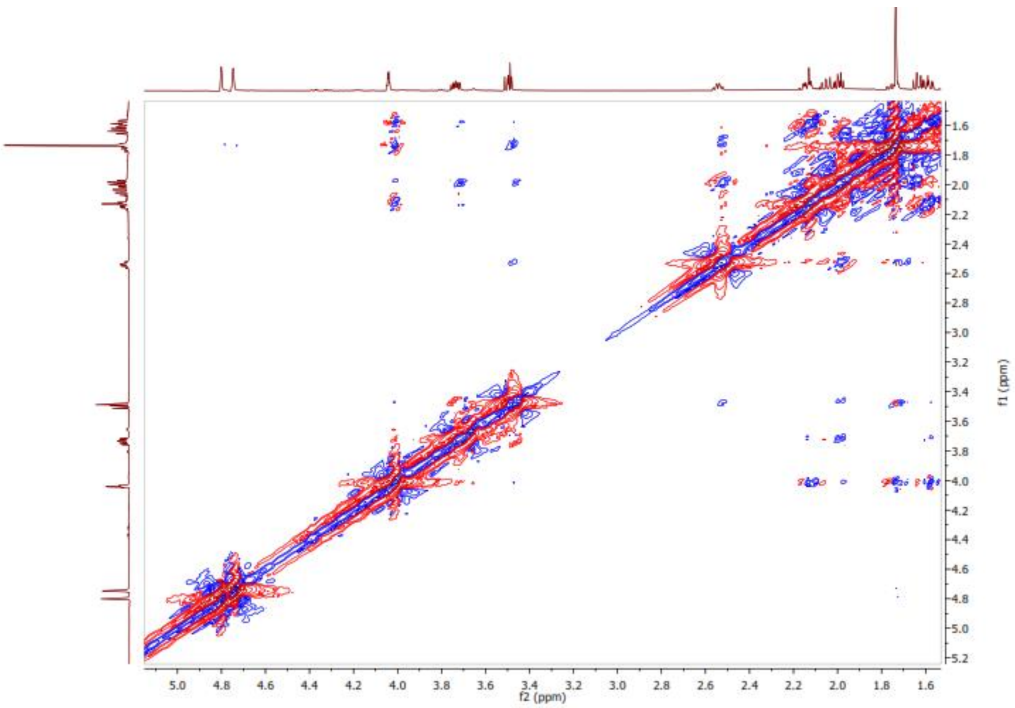

A6. NOESY spectrum of compound **1** ($CDCl_3$, 400 MHz)

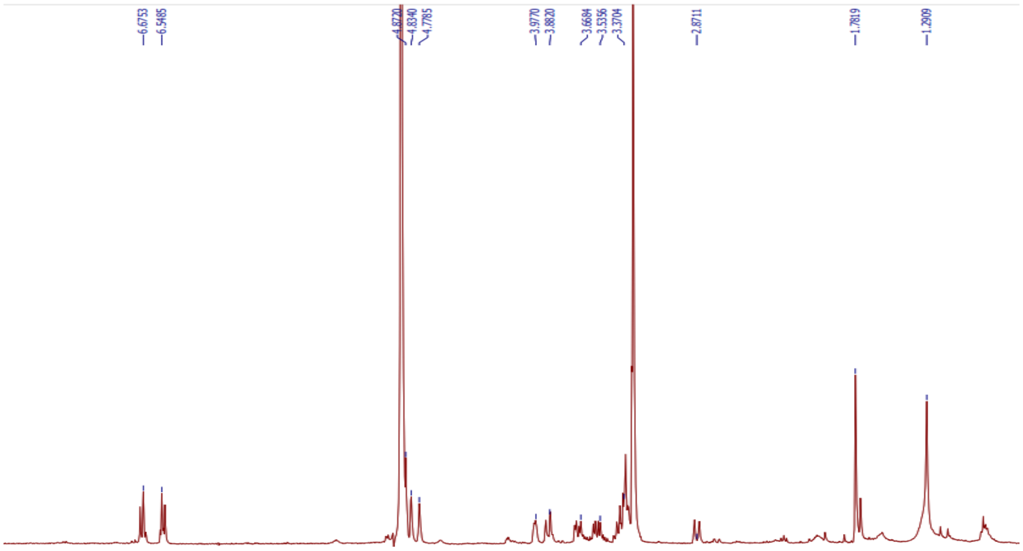

B1. ^{1}H spectrum of compound **2** (CD_3OD, 400 MHz)

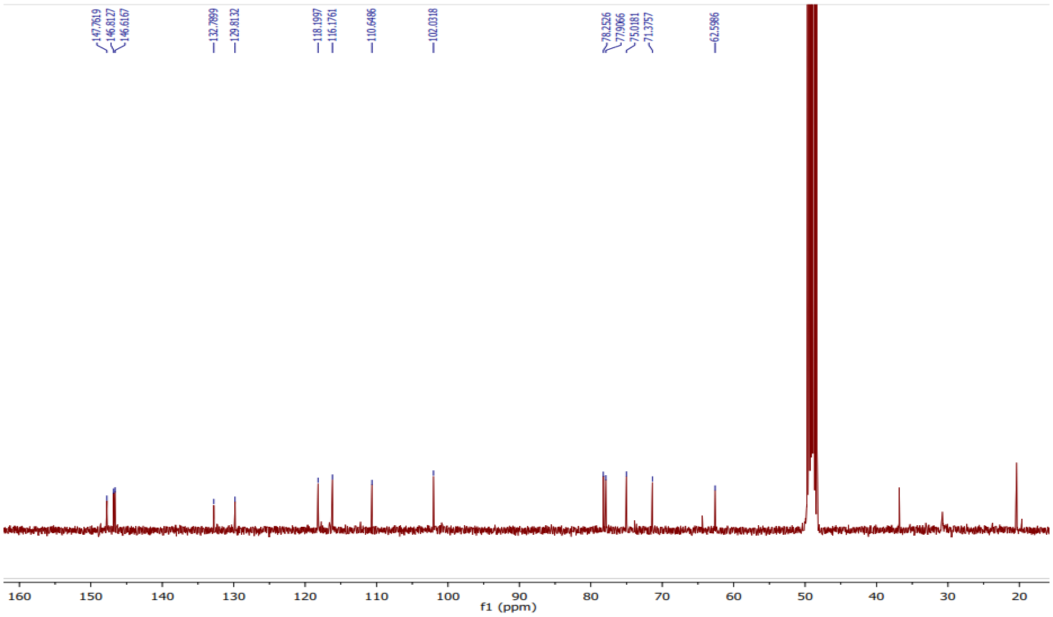

B2. ^{13}C spectrum of compound **2** (CD_3OD, 100 MHz)
